# Rule-based expert system to assess caving output ratio in top coal caving

**DOI:** 10.1371/journal.pone.0238138

**Published:** 2020-09-04

**Authors:** HaiYan Jiang, Qinghui Song, Kuidong Gao, QingJun Song, XieGuang Zhao

**Affiliations:** 1 Tai-an School, Shandong University of Science & Technology, Tai-an, Shandong, China; 2 Department of Mechanical and Electronic Engineering, Shandong University of Science & Technology, Qingdao, Shandong, China; 3 Shandong Province Key Laboratory of Mine Mechanical Engineering, Shandong University of Science & Technology, Qingdao, Shandong, China; Universita degli Studi del Molise, ITALY

## Abstract

Coal mining professionals in coal mining have recognized that the assessment of top coal release rate can not only improve the recovery rate of top coal, but also improve the quality of coal. But the process was often performed using a manual-based operation mode, which intensifies workload and difficulty, and is at risk of human errors. The study designs a assessment system to give the caving output ratio in top coal caving as accurately as possible based on the parameters adaptive Takagi-Sugeno (T-S) fuzzy system and the Levenberg-Marquardt (LM) algorithm. The main goal of the adaptive parameters based on LM algorithm is to construct its damping factor in the light of lowering of the objective function which is as taken as the index of termination iteration. The performance of the system is evaluated by Pearson correlation coefficient, Coefficient of Determination and relative error where the results of the Takagi-Sugeno method and the parameters adaptive Takagi-Sugeno method are compared to make the evaluation more robust and comprehensive.

## 1. Introduction

Fully mechanized top-coal caving is one of the most important production technologies to realize high production, high efficiency and low-consumption coal mining. Therefore, the research on automation of top-coal caving is of great significance to the development of coal industry. Some of the previously developed coal-rock recognition methods for automation of top-coal caving include the gamma-ray method [1], vibration detection method [2], acoustic detection method [3], and multi-sensor detection method [4–6], whereas the previously developed methods are aimed at identifying the state of coal-rock mixed in the process of top coal caving. As we well know, one of the focuses of scientific and technological developments in fully mechanized top coal caving system is related to improving the assessment of caving output ratio. The aforementioned discussion motivates us to develop a method to analyze the relationship between the caving output ratio (COR) and rock proportion of coal-rock flow (RPCRF), so as to assess the COR and meet the embedded equipment requirements of coal caving automation platform.

Artificial intelligence (AI) and increased computing power have long held the promise of improving assessment and prognostication [7]. Expert systems are a branch of applied AI, and were developed by the AI community in the mid-1960s [8]. Rule-based Expert System is the simplest form of AI, which uses rules as the representation for encoding knowledge from a fairly narrow area into an automated system [9]. In this study, the Takagi-Sugeno (T-S) fuzzy system was chosen, because it can solve complex and high-dimensional problems relying only on a few rules. T-S fuzzy systems proposed by Takagi and Sugeno in 1985 are an effective tool for approximation of uncertain nonlinear systems based on fuzzy if-then implication rules, and each rule refers to a local linear system, so the T-S fuzzy model approximates the original nonlinear system [10,11]. T-S model is very powerful tool which is implemented in many prediction or assessment fields, like transport [12], energy [13], chemical industry [14,15], aerospace [16] etc.

Some of the previously applications include the time-based optimization methods [17,18], and the rules-based optimization methods [19–21]. All of the aforementioned methodologies with T-S fuzzy are based on either optimization of fuzzy rule, or robust finite time. As everyone knows, one of the most important things is parameter identification of fuzzy rule in the framework of T-S fuzzy system. The LM method was applied as a parameter optimization method for T-S fuzzy rule [19]. Derakhshandeh, et al. applied LM approach for solving the power flow problems in the ill-conditioned power systems [22,23]. Dkhichi, et al. used LM combined with simulated annealing to identify parameters of solar cell model [24]. In the literature [25], the LM algorithm was used to optimize multi-hidden-layer wavelet neural network model which estimated the state of charge of lithium-ion batteries and had better performance on estimation accuracy and applicability. The LM algorithm was employed to adjust the nominal parameters of PV cell/modules, the model based LM method showed the best potential for the assessment of PV modules behaviors under dynamic weather conditions and even in real time operation, and the error between the measured and the simulated outputs was minimized [26].

It can be seen that LM approach is widely used in parameter identification. However, when the updating factor of LM is a constant value, it is difficult to improve the convergence rate of nonlinear system. The aforementioned discussion motivates us to develop a method to find an appropriate value for the updating factor of LM. Additionally, using the approximate optimum value, the parameters or functions in the consequence of the T-S fuzzy system are determined.

In addition, this paper is different in the following aspects: 1) Analyze the relationship between the COR and the RPCRF; 2) determine the parameters of the consequent part of a rule from LM formulation of the problem using adaptive updating factor method; 3) the expert system is constructed based on T-S fuzzy rules and LM parameter with adaptive updating factor method, and assesses the COR; 4)detailed analytical results are presented to guarantee the assessment of COR.

In the next section, the paper is organized as follows: Section 2 provides both Simulation experiment and expert system based rule as well as LM methods. In section 3, we explain the T-S fuzzy inference used to develop COR expert system. The validation method and procedure is reported in Section 4. The main conclusions and future works are given in Section 5.

## 2. Background and methods

### 2.1 Simulation experiments and data acquisition

The simulation experiments were designed to realistically simulate the actual process of top-coal caving such that the intrinsic relation between RPCRF and COR can be directly reflected in the simulation. The caving output ratio was determined by measuring the actual weight of the top-coal drawn out of the caving inserting plates or caving windows. Although the measuring actual weigh method is very accurate, it is a very particularly difficult. It is found that the weighing method is very close to the marker method in the experiment, and therefore the marker method can be used to estimate the caving output ratio. In the simulation experiment, the output ratio of different levels of top coal can be determined by calculating the released markers at the different levels [27].

**Fig 1:**
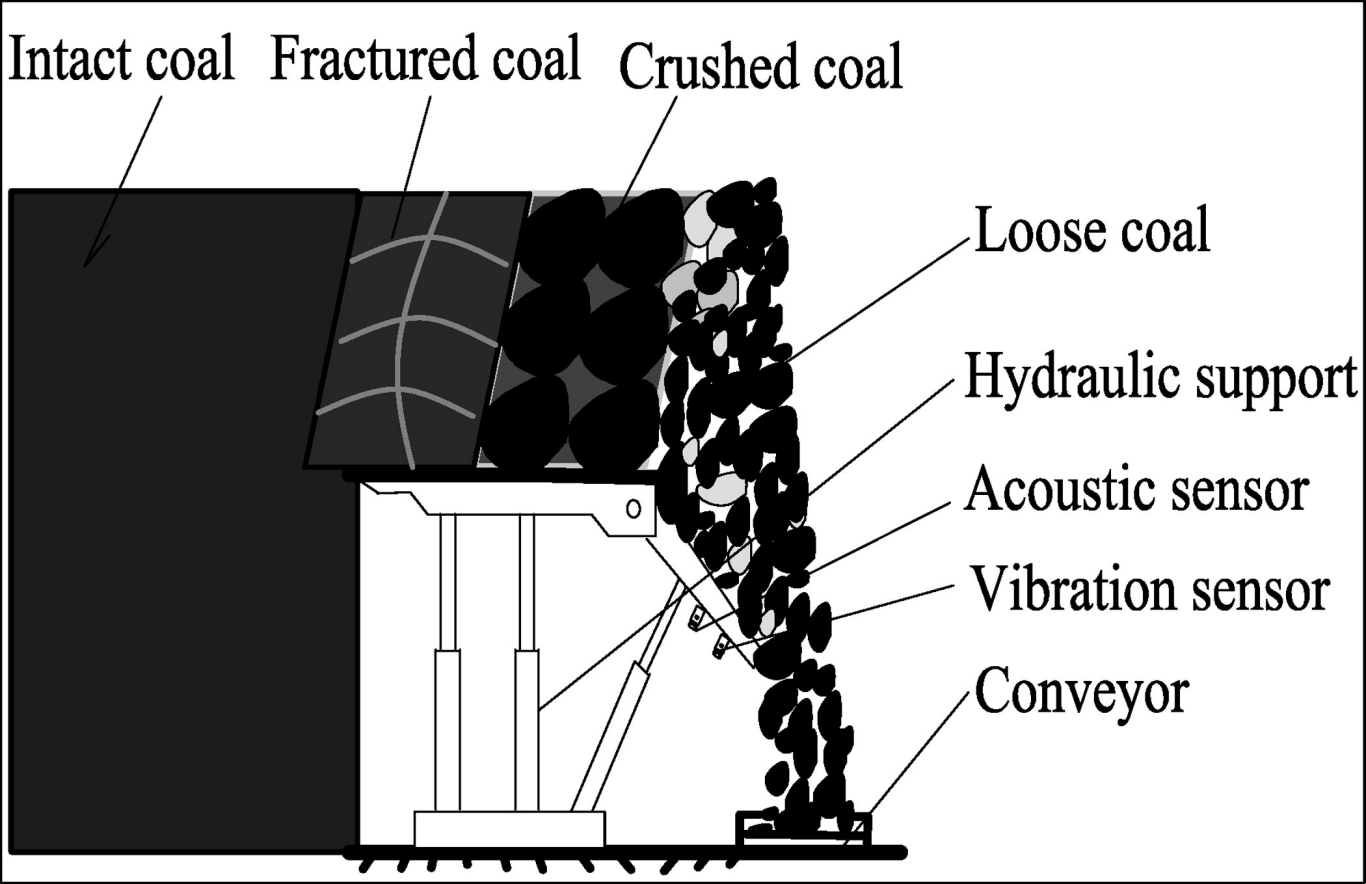
The sketch map of sensor installation.

**Fig 2:**
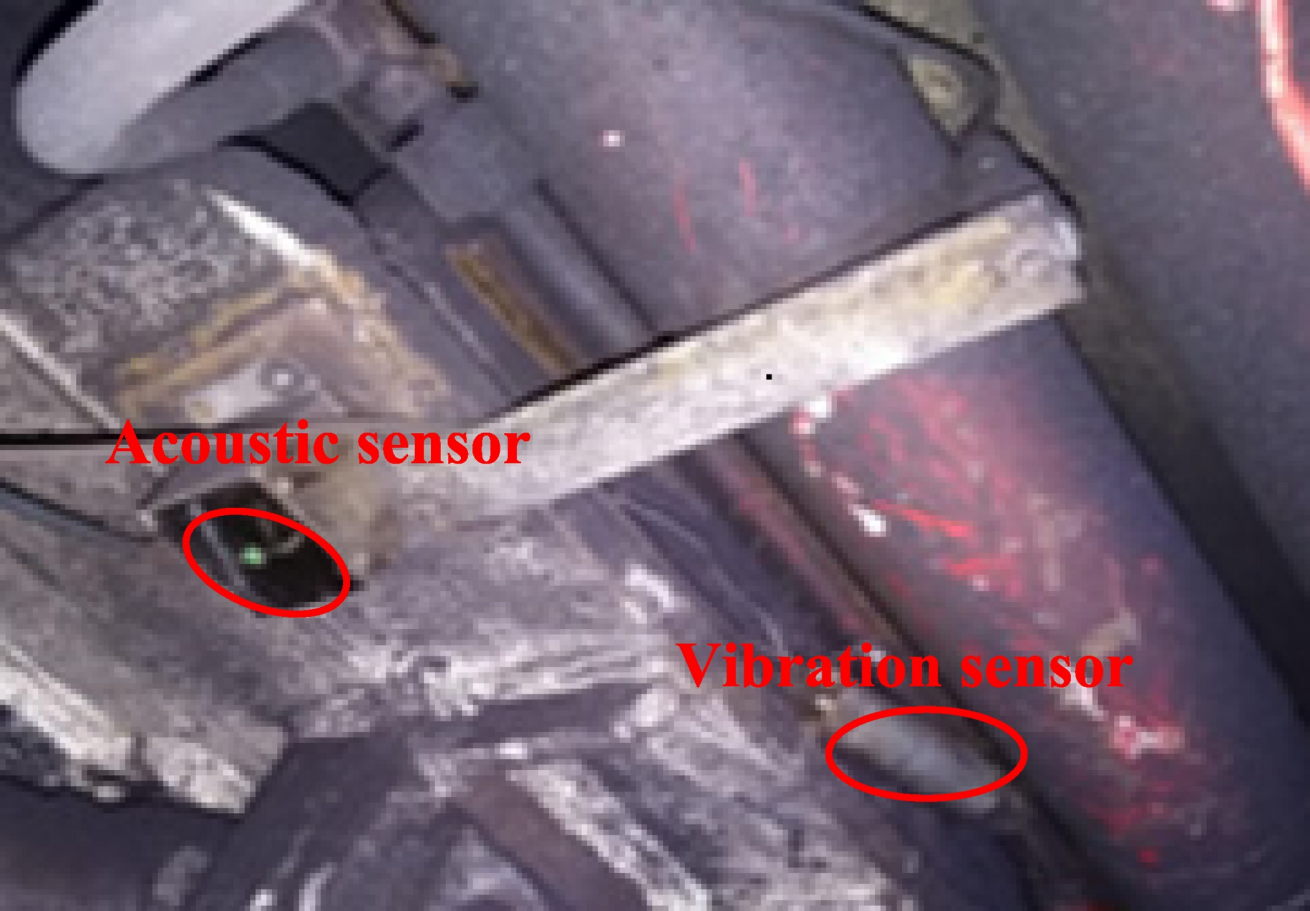
Position of sensors on the hydraulic support.

Using the coal-rock recognition idea of the multi-sensor information fusion in [5], we can obtain the more accurate RPCRF according to the sensor installation and data acquisition methods. The installation position of sensor is shown in Figs 1and 2, and the definition of rock proportion has been described in Ref [5].

### 2.2 Design expert system

The expert system designed was an inference system based on fuzzy logic for assessing COR, the inference form of if-then-else statements is enough to handle with a rather complicated area [28]. Most fuzzy controllers have been designed based on human operator experience and/or control engineer knowledge. It is, however, often the case that an operator cannot tell linguistically what kind of action it takes in a particular situation. In this respect it is quite useful to give a way to model control actions by using numerical data [10]. T-S fuzzy system can provide a reasonable framework for modeling by decomposition of a nonlinear system into a collection of local linear models. And it is one of the most used because of its good results in different areas and due to its mathematical treatability [29].

T-S fuzzy systems consist of the “if-then” rules with fuzzy antecedents and mathematical functions in the consequence part. The premise of an implication is the description of fuzzy subspace of inputs and its consequence is a linear input-output relation.

In detail, for fuzzy input variable ***x*** = (*x*_1_,*x*_2_,…,*x*_n_) ϵ R^*n*^ and *x*_1_−*x*_*n*_ are singletons, the *i-th* rule in T-S fuzzy system is presented as [10]

$$R_{i}:\;\text{if}x_{1}\text{is}{\tilde{A}}_{1}^{i},x_{2}\text{is}{\tilde{A}}_{2}^{i},\ldots ,x_{n}\text{is}{\tilde{A}}_{n}^{i},\;\text{then}$$

yi=p0i+p1ix1+p2ix2+…+pnixn(i=1,2,…,N) (1)

where *N* is the number of rules, ${\tilde{A}}_{j}^{i}$ is the linguistic variable, *x*_*j*_ is the *j-th* input, *y*_*i*_ is the output of the fuzzy rule R_*i*_, and $p_{j}^{i}$is the parameter to be identified. The fuzzy rule R_*i*_ must be complete and cover all fuzzy partitions of the input space.

Suppose that $\left\{x_{1}^{0},x_{2}^{0},\ldots ,x_{n}^{0}\right\}$ is a given input vector, the overall output of the model is computed by

$$y^{0}=\frac{\sum_{i=1}^{N}\eta_{i}y_{i}}{\sum_{i=1}^{N}\eta_{i}}$$ (2)

where *η*_i_ is the weight of the *i-th* fuzzy rule and denotes the belief in the *i-th* fuzzy rule for a given input, and it is derived as

$$\eta_{i}=\overset{n}{\underset{j=1}{\cap }}\mu_{{\tilde{A}}_{j}^{i}}(x_{j}^{0})$$ (3)

where stands for min operation and $\mu_{{\tilde{A}}_{j}^{i}}(x_{j}^{0})$ means the grade of the membership of $x_{j}^{0}$.

The normalized weighting coefficient $\delta_{i}=\eta_{i}/\sum_{i=1}^{N}\eta_{i}$ is added, so the fuzzy model output (2) can be rephrased as

$$y^{0}=\sum_{i=1}^{N}\delta_{i}y_{i}=\sum_{i=1}^{N}\left(\delta_{i}p_{0}^{i}+\delta_{i}\sum_{j=1}^{n}p_{j}^{i}x_{j}^{0}\right)$$ (4)

Although the Eq (4) is a linear combination of inputs, it can represent a highly non-linear input-output relation and imply that each rule describes local model of the system.

### 2.3 Adaptive parameter identification based on LM

In this paper, the consequence parameters $p_{0}^{i},p_{1}^{i},p_{2}^{i},\ldots ,p_{n}^{i}$(*i* =1,2,…,*N*) of T-S fuzzy system are identified by LM method based on the analysis of input-output data. The LM method was first proposed by Kenneth Levenberg and presented again by Donald Marquardt. It is an iterative non-linear least squares identification method which belongs to the continuous optimization domain. The traditional method of LM has the advantage to behave as two methods based on different orders of gradient: “steepest descent” and “Gauss-Newton” [24].

Consider the nonlinear system of equations

F(x)=0 (5)

where ***F*: R**^*n*^→**R**^*m*^ is a continuously differentiable function, we denote $F={\left(f_{1}(x),f_{2}(x),\ldots .,f_{m}(x)\right)}^{T}$and J(x)=F'(x) for all *x* ϵ *R*^*n*^.

The most common method to solve the non-linear equations is to compute the following equation in each iteration

$$d_{k}=-{\left(J_{k}^{T}J_{k}+\lambda_{k}I\right)}^{-1}J_{k}^{T}F_{k}$$ (6)

Where ***F***_*k*_ = *F*(***x***_*k*_) and ***J***_*k*_ = *F’*(***x***_*k*_) is the Jacobian of ***F*** at ***x***_*k*_. ***I*** is identity matrix and *k* is the counter of the iterations. The damping factor *λ* plays a crucial role in leading the convergence procedure of the algorithm, and its value must be determined at each iteration of LM to increment the diagonal of $J_{k}^{T}J_{k}$.

Therefore, the parameters to be identified are updated at each step of iteration using the equality

$$p_{k+1}=p_{k}+d_{k}$$ (7)

In Eq (7), the new parameter vector ***p***_k+1_ is equal to the old parameter vector ***p***_k_ plus a corrective term *d*_*k*_. It is interesting to note that the correction term ***d***_k_ contains the new data ***x*** and the damping factor *λ*. Usually *λ* is set to an updating factor by *λ* = *c*·*λ*, *c* denotes a constant value, determined by trial, the most assigned value to this constant is 10 or 10^−1^ [30]. By using the above classical method to set *λ* value, the LM algorithm does not always converge to the optimal parameters [24]. Coelho introduced a new method for determining an approximate optimum value for LM constant, and the method increases the convergence rate of nonlinear least-squares problems for objective functions [31].

For the purpose of engineering applications, especially for convenience of calculation, the objective function *S* based on the least-squares is represented as

$$S(p)=\sum_{k=1}^{M}w_{k}{\left[F_{o,k}-F_{c,k}(p)\right]}^{2}$$ (8)

where *F*_o,k_ is a set of observations from the modeling function *F*_c,k_ which is constructed through the parameters ***p***, *M* is the number of observations, *w*_k_ is the weighting of the *k-th* observation and *M* is the sum of squared differences.

Here *S* is a function that satisfies the conditions of the Taylor’s formula, and Eq (8) can be expanded with first-order expansion of Taylor’s formula at the parameter vector ***p*** to form *S*_t_:

$$S_{t}(p+\Delta p)=S(p)+\sum_{i=1}^{N}\frac{\partial S}{\partial p_{i}}\Delta p_{i}$$ (9)

If *S* is quadratic in ***p*** then *S*_*t*_(***p***+*Δ**p***) = *S*(***p***+*Δ**p***) and convergence is achieved in one nonlinear least-squares iteration. The damping factor *λ* is given by the following equation

$$\lambda_{k+1}=\{\begin{array}{cc} 10\max(\lambda_{k},10^{-1}), & (1)\Delta S\geq 0,\\ 10^{-1}\lambda_{k}, & (2)\Delta S<0\;and\;Q_{k}>5,\\ \frac{1}{2}m_{k}\lambda_{k}, & (3)\Delta S<0\;and\;m_{k}\leq 1,\\ m_{k}(\lambda_{k}+\frac{1}{2})-\frac{1}{2}, & (4)\Delta S<0\;and\;m_{k}>1, \end{array}$$ (10)

where *ΔS* = *S*(***p***+*Δ**p***) −*S*(***p***), *ΔS*_*t*_ = *S*_*t*_(***p***+*Δ**p***) −*S*(***p***), *r*_*k*_ = *ΔS*_*t*_/*ΔS*, *m*_*k*_ = max[*r*_*k*_, 0.4], $Q_{k}=\max[\sum_{i=k-10}^{k}s_{i},0]$, and $s_{k}\{\begin{array}{cc} 1, & r_{k}>1\\ -1, & r_{k}\leq 1 \end{array}$.

Eq (10) shows the formulation of *λ*, which takes *ΔS*_*t*_/*ΔS* into account and can better follow the time-varying system. The parameter *λ* is called adaptive damping factor, since it gives an approximate optimum value for LM constant in the optimization. The adaptive factor LM method (AFLM) increases the convergence rate of nonlinear least-squares problems for objective functions that are far from quadratic [27]. It is the most important that the adaptive damping factor method of LM is applied in the parameters identification of the T-S fuzzy system consequence.

## 3. Design of proposed rule-based expert system

The expert system model is developed to assess the COR in top coal caving. The technique consists of four phases: data analysis, parameter identification, fuzzy inference and knowledge representation.

### 3.1 Data analysis

**Fig 3:**
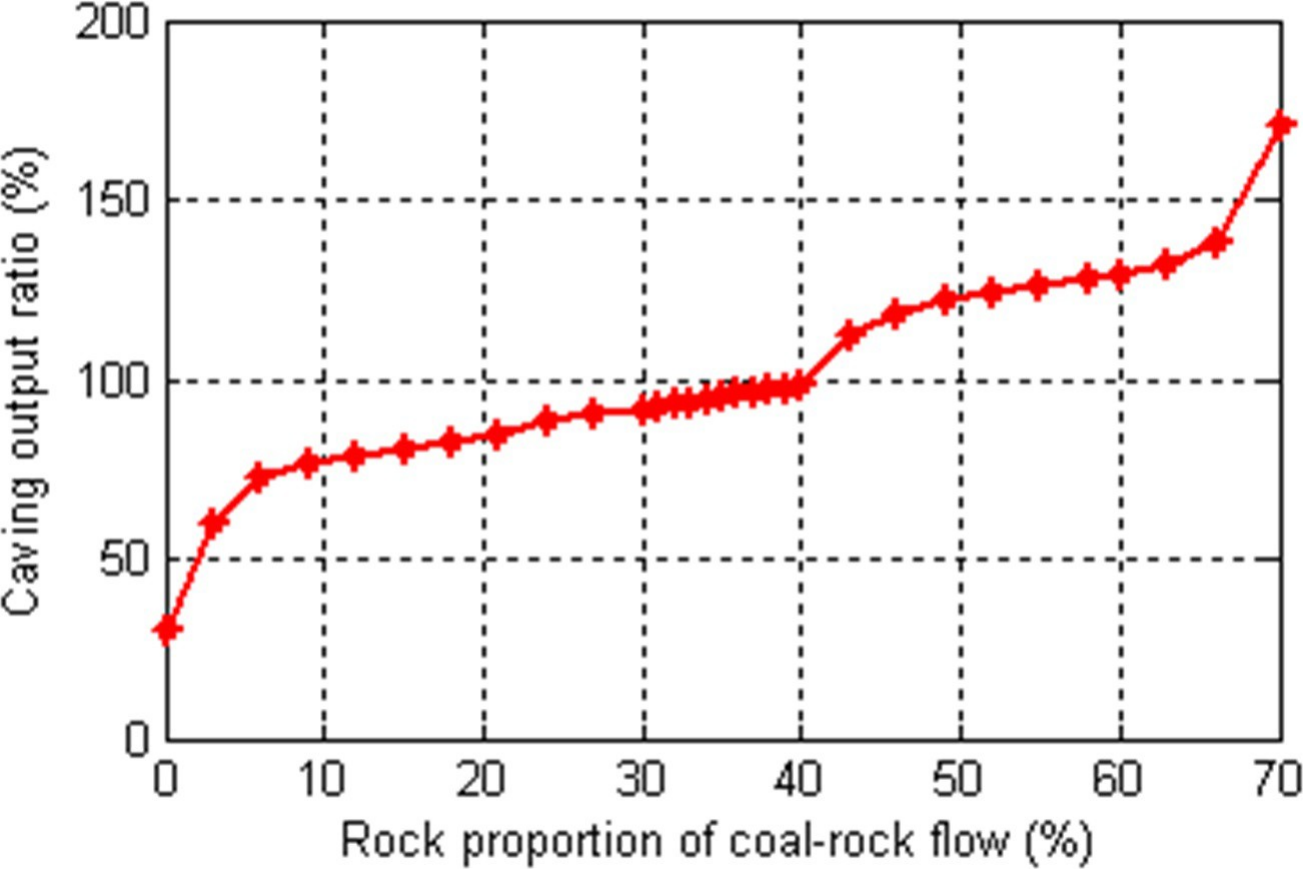
Relation between RPCRF and COR.

In order to study the relation between proportion of rock in the coal-rock flow and caving output ratio in the process of top coal caving, we conducted a large number of physical simulation experiments, i.e., “coal caving, signal extraction, rock proportion, window closing, quality statistics of top coal and rock” were carried out cyclically. The results are shown in Fig 3.

Analyzing the results depicted in Fig 3, three facts are revealed. First of all, the COR increases sharply before the RPCRF is about 4%. Second, the COR reaches 90% to 100% with stable change when the RPCRF is 30% to 40%. Finally, when the RPCRF is more than 50%, the pattern of coal-rock flow change qualitatively, then the increasing of RPCRF has a lower effect on improving COR, and it is not conducive to subsequent caving. Therefore, when the RPCRF is 30% to 40%, the caving inserting plates or caving window is closed to stop caving for producing as more coal as possible, the maximum of RPCRF should not exceed 50%, which can improve the efficiency of caving.

Therefore, at this step, the following factors were determined: the COR to be assessed as the output of the fuzzy inference system and the RPCRF to be used as its input.

### 3.2 Expert system

The presented expert system used Sugeno’s inference system to assess COR. Its base structure includes three main components: a fuzzy processor, which converts a crisp input into fuzzy values; an inference engine, which applies a fuzzy logic controller to get a crisp output; a knowledge base, which is comprised of a set of fuzzy rules and membership functions. Here we take 7 rules as an example to illustrate the design process of rule-based expert system in assessing COR. The inference module was implemented through the Fuzzy Logic Toolbox from Matlab software version R2013a according the Sugeno method.

#### 3.2.1 Parameter identification process

According to the identification algorithm discussed in Ref. [10], seven control rules are derived that can be called a fuzzy model of operator's control, where a control rule is of the form

$$y_{i}=p_{0}^{i}+p_{1}^{i}x$$ (11)

where *y*_*i*_ denotes the COR and *x* denotes the RPCRF.

**Fig 4:**
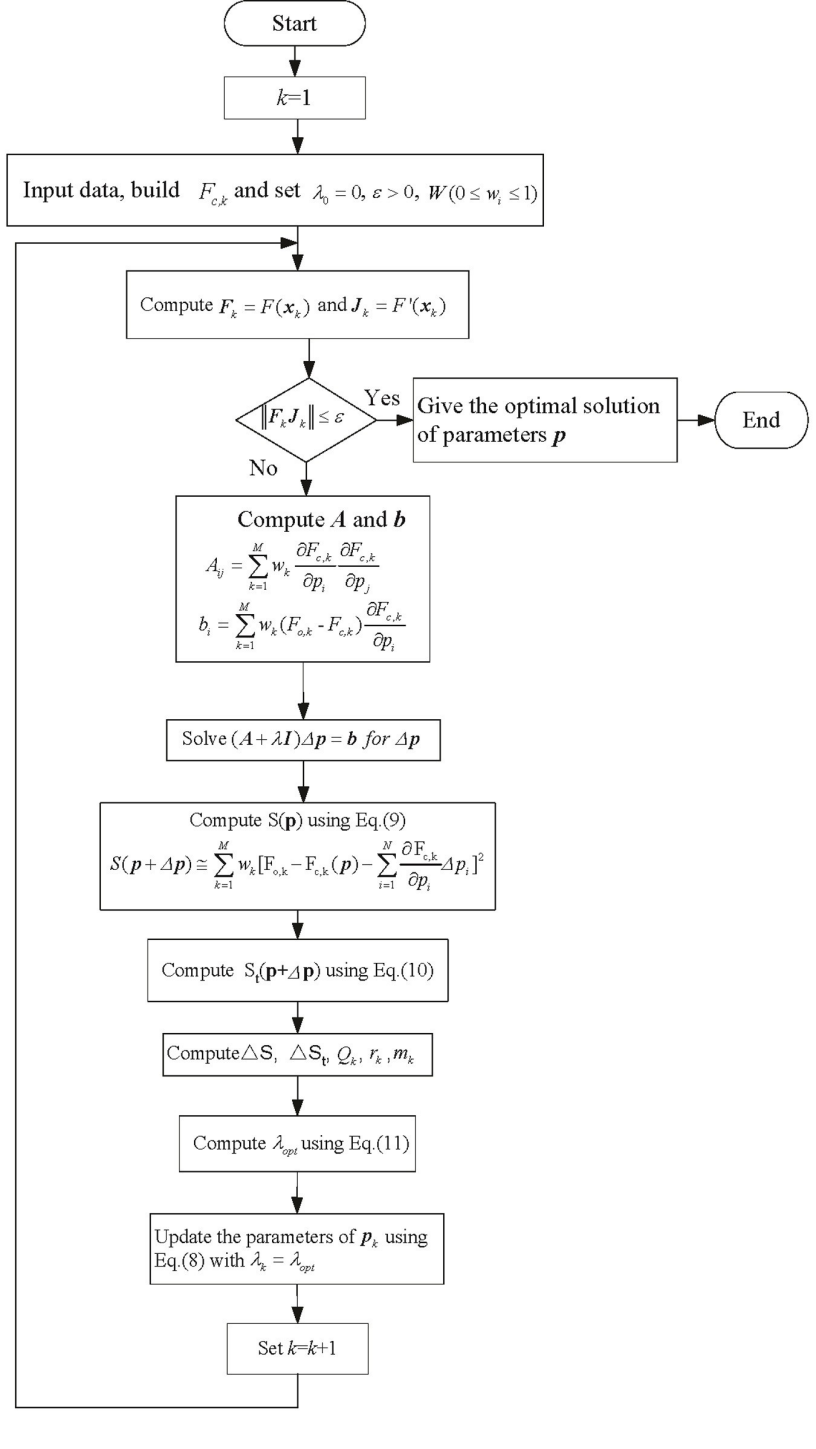
Flowchart of the AFLM algorithm for parameter identification of COR assessment model.

In order to identify the intrinsic parameters ***p***, the experimental data are fitted according to the model of Eq (11). The objective function used in the optimal fitting process is the minimization of squared between the fitting and experimental observation curves, which is given in Eq (8). The minimization of the objective function cannot be done in analytically intuitive way due to the strong nonlinear characteristic of COR. The sum of residuals squared has quadratic convergence. Hence the optimization algorithm based on the least squares principle is more appropriate to minimize such function. The application of the AFLM for solving the assessment of COR is presented in this section, and the parameter identification is presented step by step in Fig 4. The data in Table 1 indicate intrinsic parameters ***p***. There are seven rules in this case, and 14 parameters need to be optimized in the consequent as indicated in Eq (11). It is clear from Fig 4 that the 14 parameters are optimized at the same time. In order to ensure the unbiased optimization results, the 14 parameters need to be initialized similarly.

**Table 1:** Parameters *p* identified by the APLM algorithm.

Parameters	Rule1	Rule2	Rule3	Rule4	Rule5	Rule6	Rule7
p_0_	6.428329	0.779112	0.809356	0.637369	2.275546	0.803012	2.185522
p_1_	36.79599	69.07807	68.44512	73.04582	11.89838	82.19326	0.00406

Fig 4 describes in detail the process of parameter identification in the fuzzy inference output function, where ||F_*k*_J_*k*_|| is the objective function and varies with the value of parameters ***p***.

#### 3.2.2 Membership function

**Fig 5:**
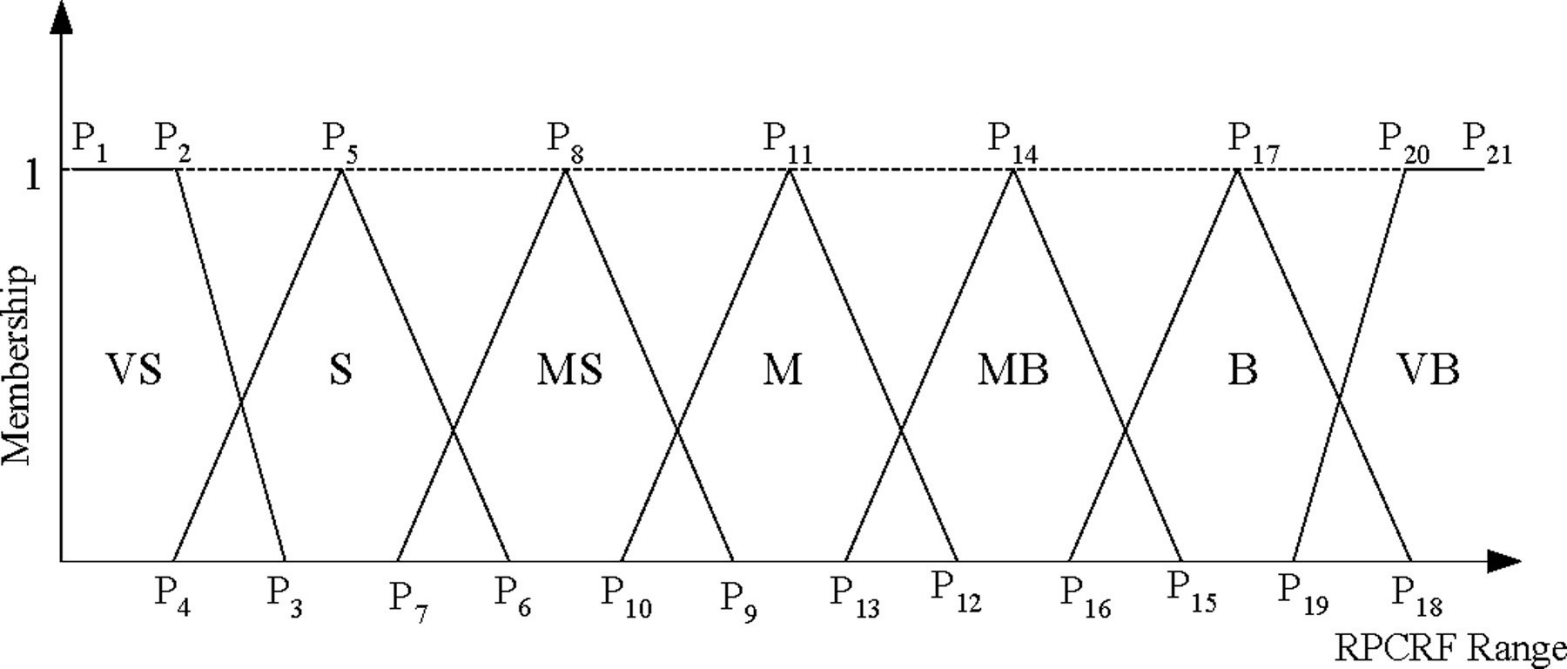
Fuzzy membership functions for RPCRF.

In fuzzy logic, each linguistic value is defined through a membership function which represents a degree to a linguistic term. In this paper we used triangular and trapezoidal shapes to represent the membership functions. Through the results of statistical analysis and an intuitive understanding of the caving output assessment, we gave the membership function shape, the domains, the degree of overlap between neighboring sets and the fuzzy sets. The membership functions shown in Fig 5 are related to the rock proportion of coal-rock flow. They are composed of seven fuzzy terms Very Small (‘VS’), Small (‘S’), Medium Small (‘MS’), Medium (‘M’), Medium Big (‘MB’), Big (‘B’) and Very Big (‘VB’). Trapezoidal membership function is used to stand for the lower and higher values and triangular membership function is used to stand for the medium value.

As shown in Fig 5, three membership points are needed to describe each membership function and hence a total of twenty-one membership points (P_1_, P_2_, …, P_21_) are required to encode a input RPCRF. In these points, first and last points are fixed as they express the minimum and maximum value of an input RPCRF. The numerical values of twenty-one membership points shown in Table 2 were applied to elaborate the membership function presented in Fig 5.

**Table 2:** Types of membership function and inflection point parameters.

Range of RPCRF	Fuzzy sets	Membership function	Inflection point parameters
[0,70]	VS	Trapezoid	[0, 3, 7]
	S	Triangle	[4, 11.5, 19]
	MS	Triangle	[15, 22.5, 30]
	M	Triangle	[25, 32.5, 40]
	MB	Triangle	[35, 42.5, 50]
	B	Triangle	[45, 52.5, 60]
	VB	Trapezoid	[55, 62.5, 70]

The number 0, 3, 7 and so on in Table 2 were the numbers that represent the points P_1_, P_2_ and P_3_ and so on respectively.

**Fig 6:**
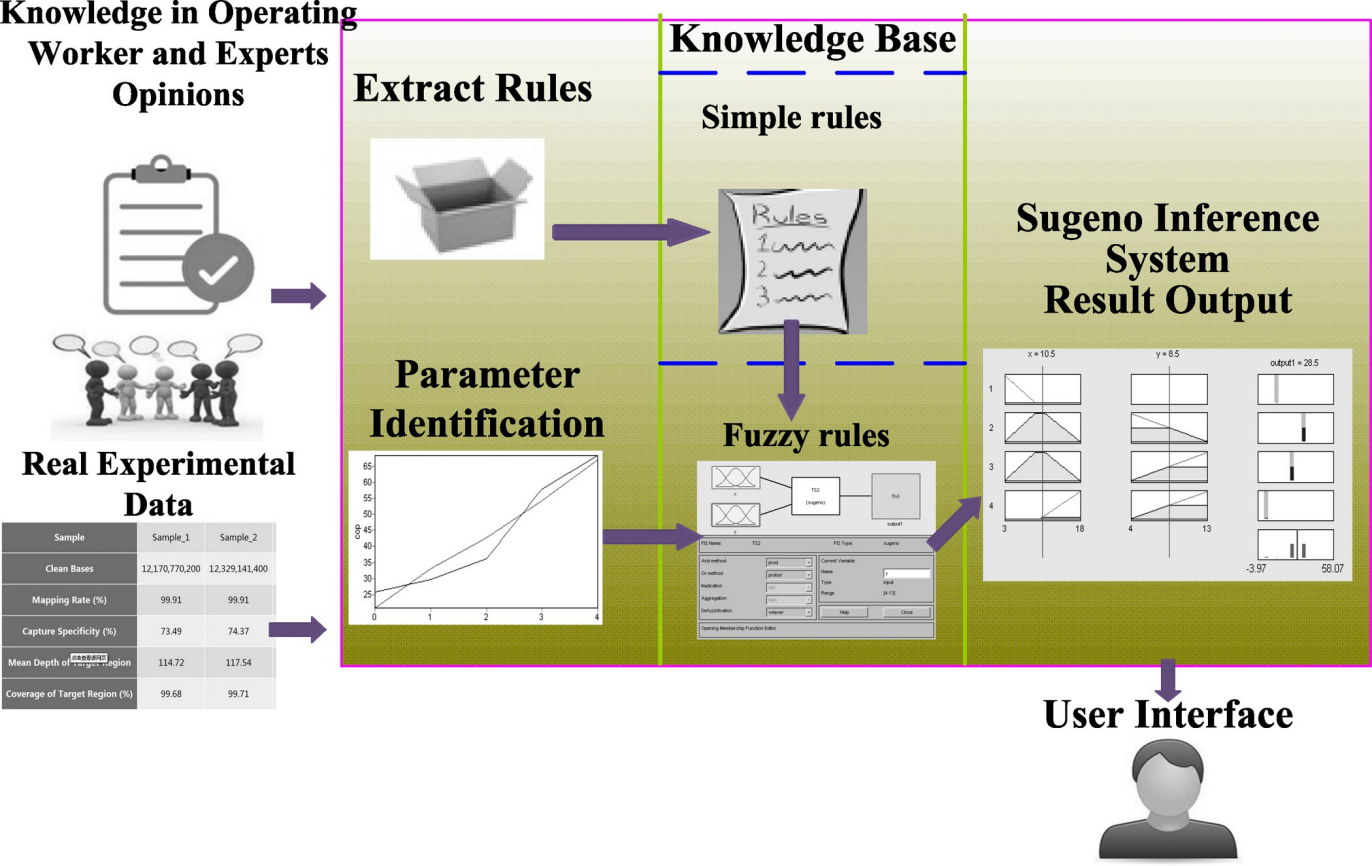
Knowledge representation in expert system of COR assessment.

**3.2.3 Knowledge representation.** A very popular method for the representation of knowledge is the use of production rules of the form “IF conditions, THEN conclusion”. The input is RPCRF and the outcome is COR. Fig 6 shows the process of knowledge representation.

Fig 6 explains parameter identification, knowledge extraction, representation, and application in the expert system of COR. The extracted rules were created in the rule-based engine and used to assess the COR. To design the rule-based engine, the results of the fuzzy rules were displayed to define the relation between the input and output. Table 3 shows the rules list.

**Table 3:** Set of fuzzy rules for the rule-based expert system.

Rule1	If *x* is VS then $y=p_{0}^{1}+p_{1}^{1}x$
Rule2	If *x* is S then $y=p_{0}^{2}+p_{1}^{2}x$
Rule3	If *x* is MS then $y=p_{0}^{3}+p_{1}^{3}x$
Rule4	If *x* is M then $y=p_{0}^{4}+p_{1}^{4}x$
Rule5	If *x* is MB then $y=p_{0}^{5}+p_{1}^{5}x$
Rule6	If *x* is B then $y=p_{0}^{6}+p_{1}^{6}x$
Rule7	If *x* is VB then $y=p_{0}^{7}+p_{1}^{7}x$

**Fig 7:**
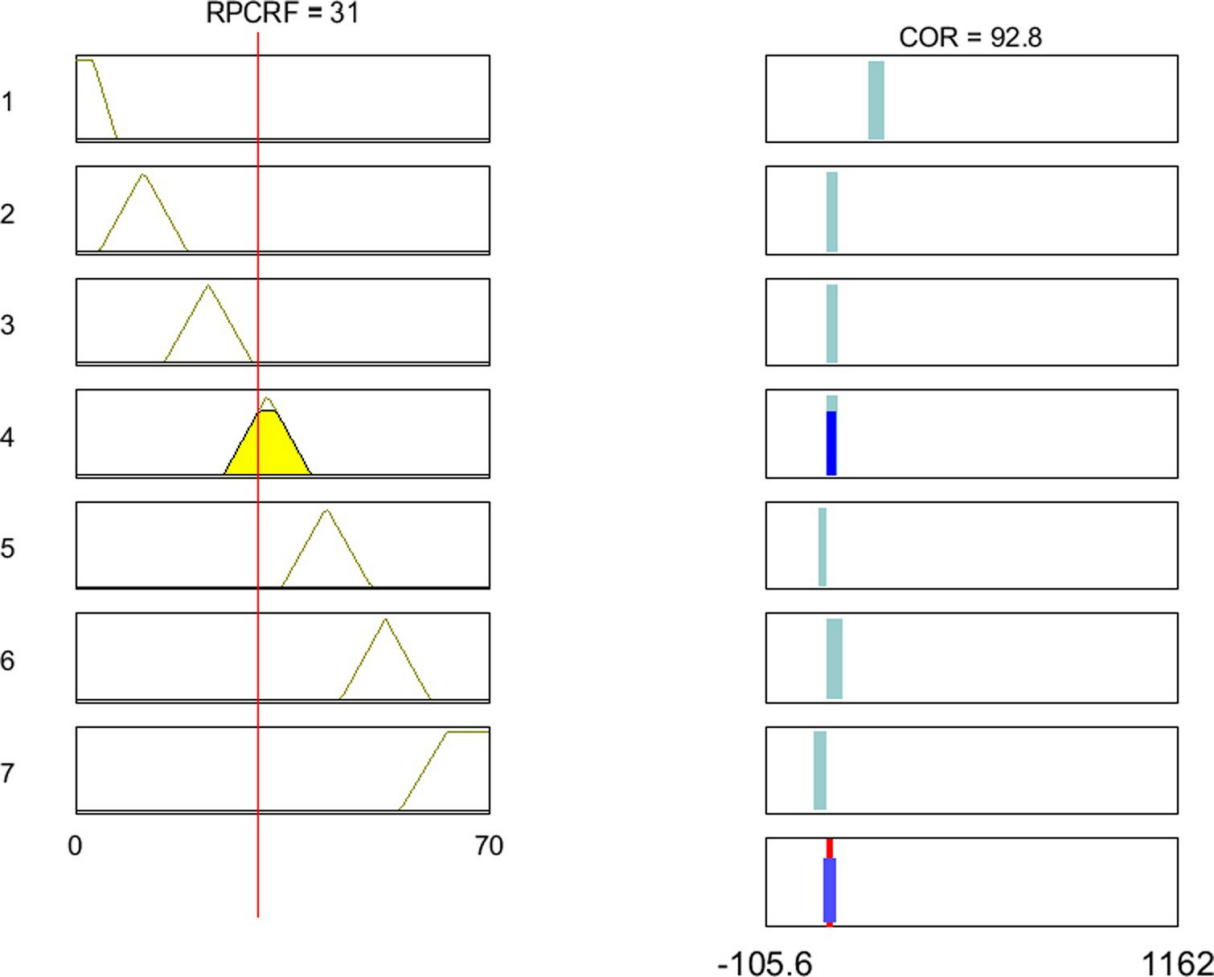
Rule structure of proposed expert system.

In Table 3, $p_{0}^{i}$ and $p_{1}^{i}$ (*i* = 1,2,…,7) are parameters ***p*** identified by the AFLM algorithm which were listed in Table 1. Fig 7 shows an example of the activation of the rules, which allows us to interpret how the expert system of COR works.

Results in Fig 7 suggest that the T-S fuzzy reasoning model is able to assess the COR according to the rock proportion of coal-rock flow. Although the above process is only the application of general T-S model, this result encourages us to continue to study a parameter adaptive T-S methodology to improve the system performance.

## 4. Validation and results

The assessment performance of the T-S expert system with APLM method is investigated under the three issues: Pearson correlation coefficient, Coefficient of Determination and relative error (%). It can be seen from Fig 3 that the function changes rapidly in the interval [0, 7] and [45, 70], respectively. The number of rules is added in these two intervals to produce different T-S expert system with different rules (e.g., 7-rule, 10-rule, 12-rule and 13-rule).

### 4.1 Validation measures

The performance of the model in assessing COR defined in Eqs (12)—(13). The Pearson correlation coefficient (*r*) measures the strength of linear association between measured and assessed values. By comparing the covariance of the measured COR(*Y*) to the assessed COR (*X*), the Pearson correlation coefficient can be expressed as

$$r=\frac{\sum_{i=1}^{N}\left(X_{i}-\bar{X})(Y_{i}-\bar{Y}\right)}{\sum_{i=1}^{N}{\left(X_{i}-\bar{X}\right)}^{2}\sum_{i=1}^{N}{\left(Y_{i}-\bar{Y}\right)}^{2}}$$ (12)

where $\bar{X}$ and $\bar{Y}$ are sample means for ***X*** and ***Y***, respectively. The value of “1” defines a perfect positive correlation and the value of “-1” defines a perfect negative correlation.

As guaranteed when the law of large numbers can be applied, *r* is a consistent estimate of the population correlation coefficient as long as the sample means, variances, and covariance are consistent [32]. Therefore, it is reasonable to use *r* to evaluate the performance of the assessment model.

Coefficient of Determination (*R*^2^) is a descriptive measure for the goodness of fit of assessment and forecasting [33] and it is calculated as

$$R^{2}=\frac{\mathrm{SSR}}{\mathrm{SST}}=\frac{\sum {\left(X-\bar{Y}\right)}^{2}}{\sum \left(Y-\bar{Y}\right)}$$ (13)

where SSR is Regression Sum of Squares, SST is Total Sum of Squares. The value of *R*^2^ is bounded between 0 and 1, such that the values close to one imply a good fit of the regression.

### 4.2 Results and discussions

**Fig 8:**
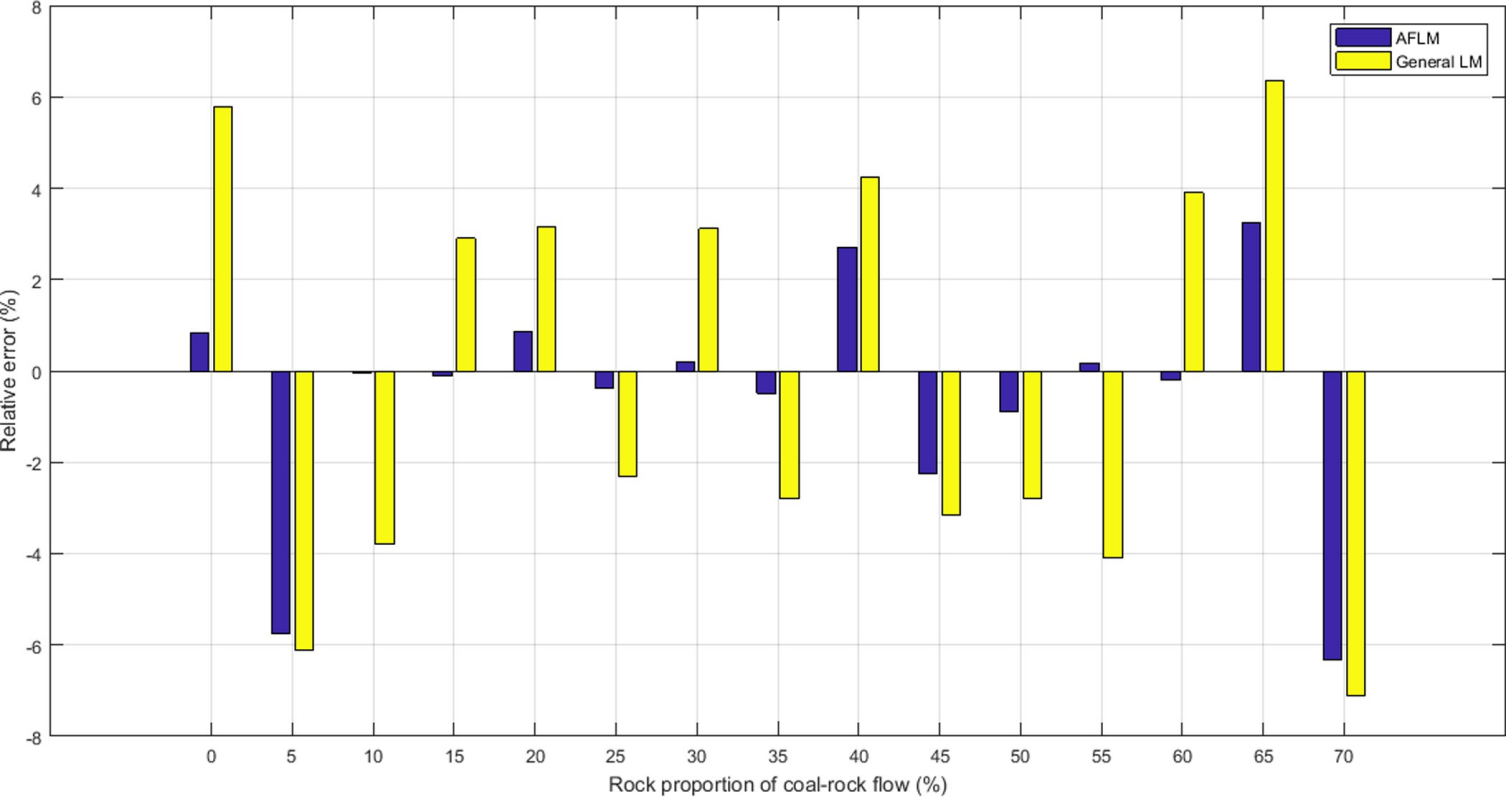
Relative error of expert system assessments.

First of all, a comparative experiment between the general LM method [19, 34] and APLM was carried out to have a clear profile. For the sake of direct comparison with the general LM, the APLM approach is applied to the same experimental data and T-S expert system with 12 rules. We show the relative error (%) of assessments and experimental observations, as plotted in Fig 8. The positive error indicates overestimation, while a negative value means underestimation.

As stated before, AFLM approaches could do well in consequence parameter identification of T-S fuzzy model, which can also be concluded in Fig 8. It is evident that the AFLM method was by far more accurate than the general LM method in terms of their simulation accuracy of the relative error criteria. In the process of top coal caving, the RPCRF is best controlled at 30%-40%, and the COR can reach 90%-100%. As can be seen from Fig 8, the AFLM approaches is able to achieve a better and more stable assessment accuracy among 30%-40% of the RPCRF. These results are confirmed by Fig 10.

**Fig 9:**
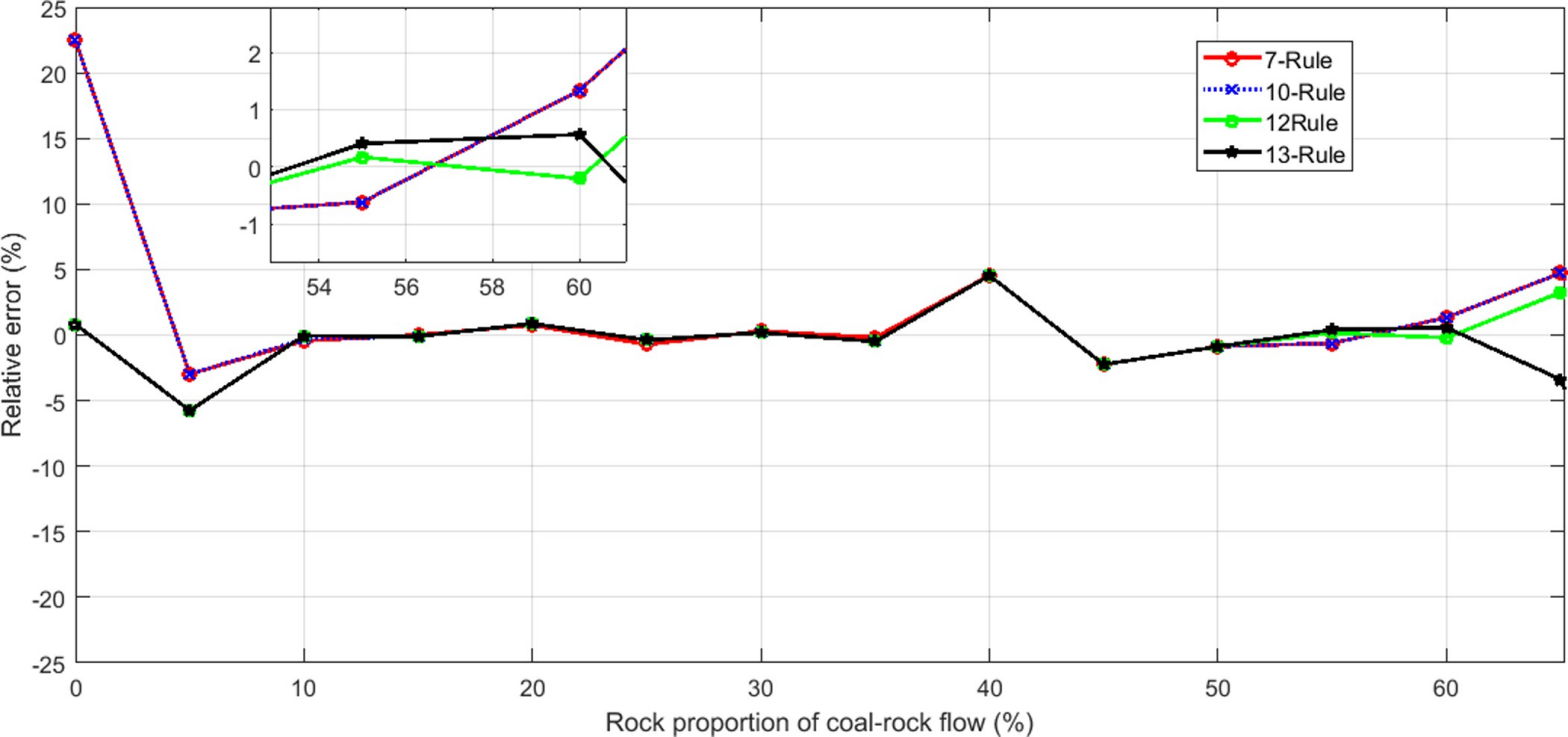
Relative error of in each T-S expert system.

In order to take a close look at the assessment accuracy obtained using different control rules under the same experimental conditions, the performance of different T-S fuzzy models with different rules was analyzed. Fig 9 demonstrates the relative errors (%) for different T-S expert systems by means of the change in the number of rules.

By observing Fig 9, it can be found out that the AFLM approaches can provide a similar relative error under four different fuzzy rules, but in the place where the curve changes steeply, the T-S expert system with 12 rules can provide a smaller relative error value and the relative error of different RPCRF is all below 6.5%. However, when a large number of gangues emerge, the shape of coal-gangue flow changes, making the caving process unstable.Thus all assessment accuracy with different control rules is reduced. But the assessment accuracy of the expert system with 12 rules is the highest.

**Fig 10:**
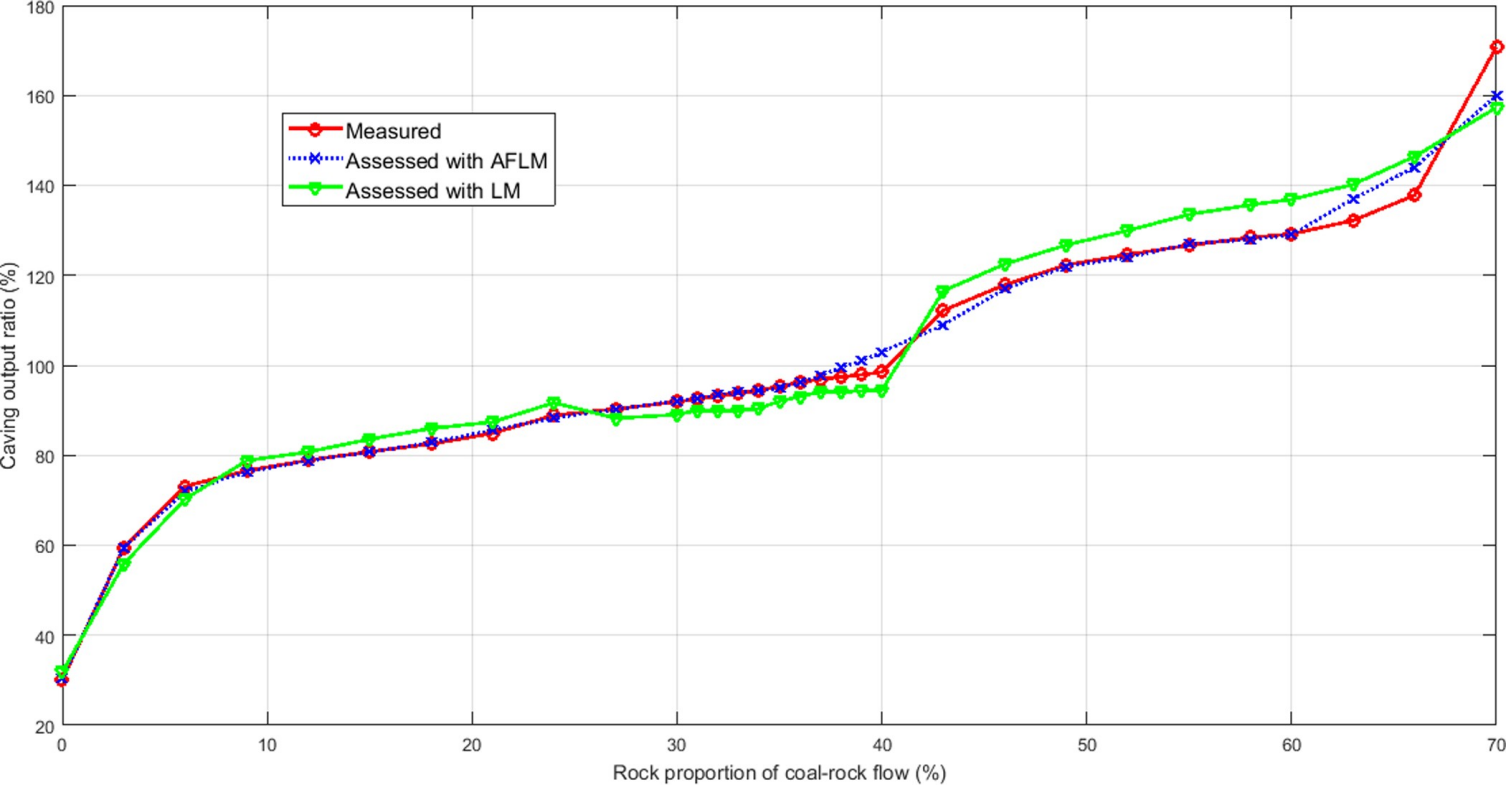
Measured COR compared with assessed COR obtained using the proposed method.

**Fig 11:**
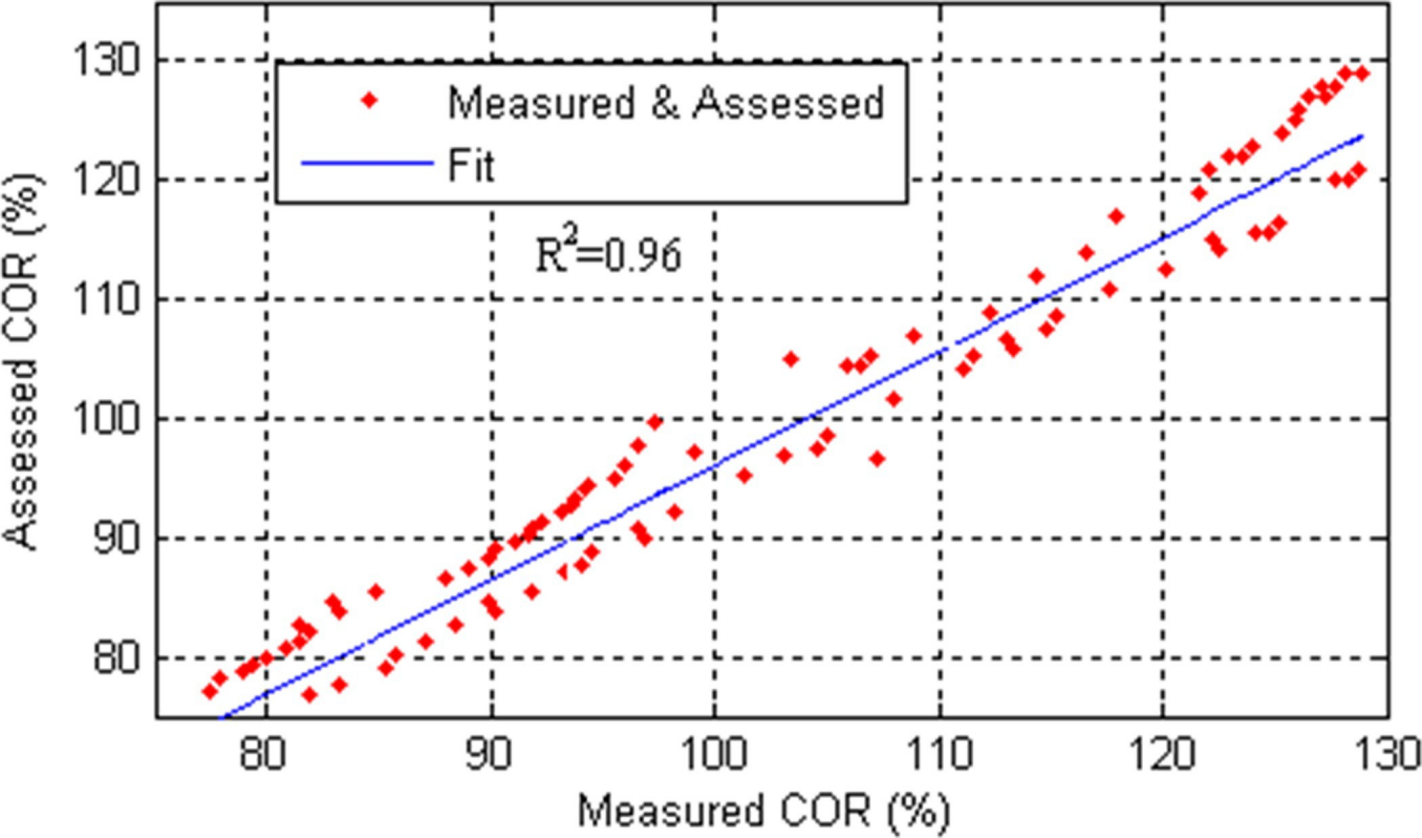
Goodness of fit between measured and assessed COR.

In order to validate the performance of T-S expert system with 12 rules, a comparison test has been carried out under the same experimental conditions. Fig 10 shows the measured curve, LM assessing curve and AFLM assessing curve, respectively. Fig 11 shows the linear correlation between measured COR and assessed COR by T-S expert system with 12 rules, which is used to evaluate the performance of the model in assessing the caving output ratio.

It is found from Fig 10 that a better agreement between the measured and assessed curves is obtained for the developed models compared to results found based on general LM approach. The validity of the proposed model lies in the ability of assessing the important events, such as 1/3 RPCRF. When the RPCRF is in the range of 30%-40%, the average assessment accuracy reaches 99.98%, whereas the overall accuracy is decreased due to complexity of caving process and measurement error. The Pearson correlation coefficient of 0.98 is obtained between measured COR and assessed COR, which shows the feasibility of the application of the T-S expert system associated with the AFLM parameter identification technique. It can also be visualized that the highest significant correlation between measured and assessed COR from Fig 11. The coefficient of determination *R*^2^ is used to measure the goodness of fit. The value of *R*^2^ is 0.96 and close to 1, which shows that the rule-based expert system can assess the trend of observed data very well. As can be seen clearly from Fig 11, the assessed data points are closer to the fitted line. Therefore the proposed system has good regression results.

According to the experimental results reported in Figs 8–11, It can be seen clearly that the proposed assessment methodology based on the adaptive factor LM algorithm and T-S fuzzy method is able to give a more accurate prediction value in the caving output ratio assessment.

## 5. Conclusions

One of the focuses of scientific and technological developments in coal automatic production systems is currently related to improving the decision making process for increasing productivity and efficiency in resource utilization. The rule-based expert systems are effective tools for the approximation of uncertain non-linear systems. In this paper, a T-S fuzzy inference system based on adaptive parameter identification method was successfully developed and applied for the assessment of COR in top coal caving work. We used the AFLM algorithm in conjunction with every newly acquired sample to identify the consequent parameters of T-S fuzzy expert system and ensure a good minimization of the objective. It was observed from the experimental results that the proposed expert system gave good results in terms of Pearson correlation coefficient, Coefficient of Determination and relative error, and showed a better agreement between the experimental and assessed curves. To our knowledge, this is the first use of a T-S inference system in the domain of assessment of the caving output ratio. Additionally, the systematic study on COR provides a good reference for the realization of unmanned coal mining. As part of our future work, an online COR assessment scheme would be developed for T-S fuzzy systems in consideration of more complex practical working conditions.

## Supporting information

S1 Data (TXT)
